# Comprehensive analysis of circRNA expression profiles and circRNA‐associated competing endogenous RNA networks in the development of mouse thymus

**DOI:** 10.1111/jcmm.15276

**Published:** 2020-04-19

**Authors:** Wenting Li, Nana Ma, Ting Yuwen, Bo Yu, Yao Zhou, Yufei Yao, Qi Li, Xiaofan Chen, Jun Wan, Yu Zhang, Wei Zhang

**Affiliations:** ^1^ Biomedical Research Institute Shenzhen Peking University ‐ The Hong Kong University of Science and Technology Medical Center Shenzhen China; ^2^ Department of Dermatology Peking University Shenzhen Hospital Shenzhen China; ^3^ Hainan Provincial Key Laboratory for human reproductive medicine and Genetic Research The First Affiliated Hospital of Hainan Medical University Hainan Medical University Hainan China; ^4^ Division of Life Science The Hong Kong University of Science and Technology Kowloon, Hong Kong China; ^5^ Greater Bay Biomedical Innocenter Shenzhen Bay Laboratory Shenzhen China; ^6^ Department of Reproductive Medicine The First Affiliated Hospital of Hainan Medical University Hainan Medical University Hainan China; ^7^ Hainan Provincial Clinical Research Center for Thalassemia The First Affiliated Hospital of Hainan Medical University Hainan Medical University Hainan China; ^8^ Key Laboratory of Tropical Translational Medicine of Ministry of Education Hainan Medical University Hainan China

**Keywords:** ceRNA network, circRNA, thymus development

## Abstract

The thymus plays an irreplaceable role as a primary lymphoid organ. However, the complicate processes of its development and involution are incompletely understood. Accumulating evidence indicates that non‐coding RNAs play key roles in the regulation of biological development. At present, the studies of the circRNA profiles and of circRNA‐associated competing endogenous RNAs (ceRNAs) in the thymus are still scarce. Here, deep‐RNA sequencing was used to study the biological mechanisms underlying the development process (from 2‐week‐old to 6‐week‐old) and the recession process (from 6‐week‐old to 3‐month‐old) of the mouse thymus. It was found that 196 circRNAs, 233 miRNAs and 3807 mRNAs were significantly dysregulated. The circRNA‐associated ceRNA networks were constructed in the mouse thymus, which were mainly involved in early embryonic development and the proliferation and division of T cells. Taken together, these results elucidated the regulatory roles of ceRNAs in the development and involution processes of the mouse thymus.

## 1 INTRODUCTION

The thymus is defined as a primary lymphoid organ with its inimitable role in T cell maturation, education and selection.^1^ It is the first formed lymphoid organ, but may also the most rapidly ageing tissue in the body.^2^ In response to the demand for large numbers of mature T cells, the thymus grows fast in the early stages of post‐natal life and reaches a plateau at 4‐6 weeks of age in the mouse.^3^ Afterwards, it starts the ageing process, of which the typical phenotype is thymic involution. The significantly decreased size and structural complexity of the thymus with age were manifested by the loss of thymocytes and thymic epithelial cells and the increase in the content of adipocytes.^4^ Actually, the processes about the rapid development and gradual involution of the thymus are still unclear.^3^

MicroRNAs (miRNAs) are single‐stranded (average size 22 nt) non‐coding RNAs that regulate gene expression at the post‐transcriptional level.^5^ Through their broad effects on gene expression, miRNAs participate in the regulation of many cellular processes such as organismal development,^6^, ^7^ cellular differentiation and apoptosis^8^, ^9^ as well as mitochondrial metabolism.^10^ MiRNAs also take part in many aspects of T cell immunity such as T cell maturation, differentiation, activation and ageing. ^11^ For example, some miRNAs including miR‐155, miR‐181c, miR‐9 and miR‐31 can regulate T cell activation by modulating the IL‐2 signalling pathway.^12^ Moreover, miR‐181a‐5p and miR‐195a‐5p expressed in thymic epithelial cells are involved in thymus involution, which directly target transforming growth factor β receptor 1 (*Tgfbr1*) and Smad family member 7 (*Smad7*) respectively.^13^, ^14^

Circular RNAs (circRNAs) are a class of single‐stranded closed RNA molecules without 3′‐poly (A) and 5′‐cap structures, which have been widely found in plants, animals and human beings.^15^ The cell‐type‐specific, tissue‐specific or developmental stage‐specific expression profiles of circRNAs suggest their regulatory functions in biological processes.^15^, ^16^ Numerous studies have reported that circRNAs are closely related to the tumorigenesis,^17^, ^18^ neurodegenerative diseases,^19^ cardiovascular diseases^20^ and immune diseases.^21^, ^22^ Some circRNAs might be involved in post‐transcriptional regulation by functioning as ‘sponges’ of miRNAs. Therefore, the circRNA‐miRNA‐mRNA networks may influence multiple biological pathways.^23^ At present, integrative analysis of circRNA‐associated competing endogenous RNAs (ceRNA) networks in the development and involution of the thymus are still scarce.

To gain further insight into the molecular events associated with thymic development and involution, RNA‐seq data were systematically analysed to identify aberrantly expressed circRNAs, miRNAs and mRNAs among mice thymuses at 2, 6 weeks and 3 months, respectively. In addition, the circRNA‐miRNA‐mRNA networks were constructed. This is the first comprehensive high‐throughput sequencing analysis of circRNA, miRNA and mRNA expression profiles in the thymus, which deepen our understanding of thymic development and involution.

## 2 MATERIALS AND METHODS

### 2.1 Animal tissues preparation

Male C57BL/6 mice were purchased from Beijing HFK Bioscience CO. LTD and maintained in a specific‐pathogen‐free environment. The mice were provided free access to standard diet until they met age requirements (2 weeks, 6 weeks and 3 months). Two biological replicates at each time point were used for sequencing. All the animal study protocols were approved by the Committee for the Ethics of Animal Experiments, Shenzhen Peking University‐The Hong Kong University of Science and Technology Medical Center (SPHMC; protocol number 2011‐004). The thymuses harvested from each group were placed in cryopreservation tubes and immediately immersed in liquid nitrogen (−196°C) for preservation.

### 2.2 RNA extraction

Total RNA from each thymus sample was isolated with the TRIzol Reagent kit (Invitrogen) and RNA degradation and contamination were monitored on 1% agarose gels. NanoPhotometer spectrophotometer (IMPLEN) was used to check the RNA purity. RNA concentration was measured with the Qubit RNA Assay Kit in Qubit 2.0 Flurometer (Life Technologies), and RNA integrity was assessed using the RNA Nano 6000 Assay Kit of the Bioanalyzer 2100 system (Agilent Technologies). All the RNA samples had an RNA Quality Index ≥8.

### 2.3 RNA‐sequencing and miRNA‐sequencing

Details of the RNA‐seq and miRNA‐seq methods are described in Table S15.

### 2.4 Differential expression analysis

The differentially expressed circRNAs (DEcircRNAs), mRNAs (DEmRNAs) and miRNAs (DEmiRNAs) were identified using DESeq Software Packages (http://bioconductor.org/packages/release/bioc/html/DESeq.html). *P* value and log_2_FC were used to screen differential transcripts with *P* < .05 and |log_2_FC|>1.

### 2.5 Integrated analysis of circRNAs‐miRNAs‐mRNAs

CircRNAs were blasted against the circBase for annotation. Those cannot be annotated were defined as novel circRNAs. For circRNAs that have been annotated in circBase, the target relationship with miRNAs can be predicted by StarBase (v2.0). For novel circRNAs, three softwares Mireap, Miranda (v3.3a) and TargetScan (v7.0) were used to predict targets. For the prediction of mRNAs interacting with circRNAs and miRNAs, miRTarBase (v6.1) was used to predict mRNAs targeted by miRNAs sponge. The triple network was finally built based on the ceRNA theory and the resulting correlation of circRNAs‐miRNAs‐mRNAs can be visualized by Cytoscape 3.01.

### 2.6 KEGG enrichment analysis of differentially expressed genes

To further understand the underlying biological mechanisms and pathways of ceRNA‐related genes, Kyoto Encyclopedia of Genes and Genomes (KEGG) pathway enrichment analysis was implemented using the clusterProfiler R package.

### 2.7 Data access

All raw and processed sequencing data have been submitted to the NCBI Gene Expression Omnibus (GEO; http://www.ncbi.nlm.nih.gov/geo/) under accession number GSE139653.

## 3 RESULTS

### 3.1 Changes in thymus tissues

The age‐related changes were observed in the thymuses derived from the 2‐week‐old, 6‐week‐old and 3‐month‐old male mice (Figure S1). Consistent with the literature, the mouse thymus grows rapidly after birth, reaches the maximal size by sexual maturity and then gradually involutes.^2^ The volume of the thymus increases significantly in the 6‐week‐old mouse compared with that in the 2‐week‐old mouse. As expected, the thymus shows remarkable atrophy in 3‐month‐old mouse compared with that in 6‐week‐old mouse.

### 3.2 Identification of differentially expressed genes

**FIGURE 1:**
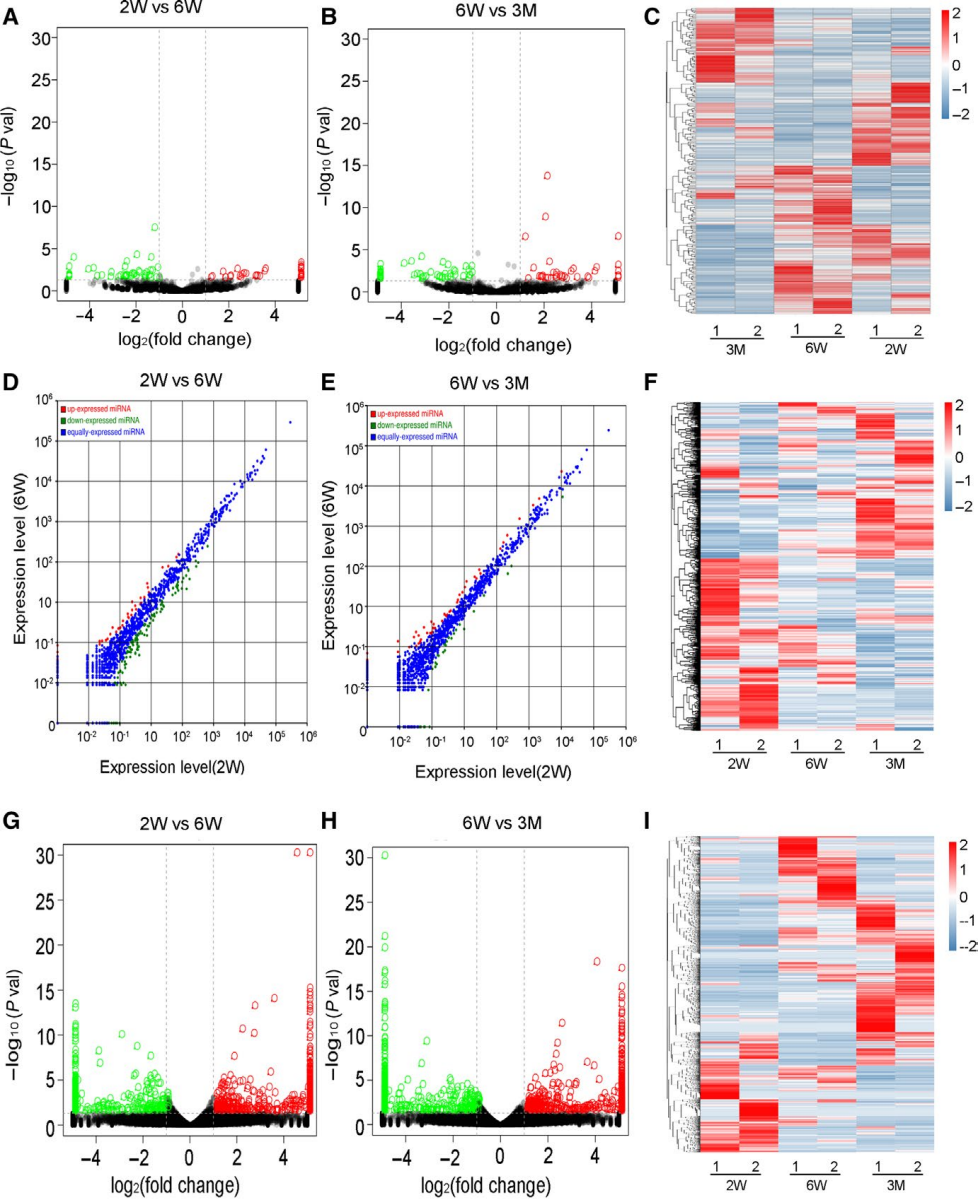
Expression profiles of distinct RNAs. A‐C, Expression profiles of circRNAs. A, B, Volcano plot of different circRNAs in the mouse thymus. A, 2W vs 6W thymus. B, 6W vs 3M thymus. Red, green and black points represent circRNAs that were up‐regulated, down‐regulated and not significantly different in the mouse thymus respectively. *x*‐axis: log2 ratio of circRNA expression levels, *y*‐axis: false‐discovery rate values (−log10 transformed) of circRNAs. C, Cluster analysis of expression of circRNAs. Red and blue: increased and decreased expression, respectively. Expression profiles are shown similarly for (D‐F) miRNAs and (G‐I) mRNAs

A total of 94 differentially expressed circRNAs (DEcircRNAs, 45 up‐regulated and 49 down‐regulated), 154 differentially expressed miRNAs (DEmiRNAs, 38 up‐regulated and 116 down‐regulated) and 1836 differentially expressed mRNAs (DEmRNAs, 1018 up‐regulated and 818 down‐regulated) were identified in the 6‐week‐old mice relative to the 2‐week‐old mice (Figure 1A,D,G). In the 3‐month‐old mice, 102 DEcircRNAs (54 up‐regulated and 48 down‐regulated), 79 DEmiRNAs (47 up‐regulated and 32 down‐regulated) and 1971 DEmRNAs (1005 up‐regulated and 966 down‐regulated) were observed relative to the 6‐week‐old mice (Figure 1B,E,H). Cluster analyses and heat‐maps about the expression of these circRNAs, miRNAs and mRNAs were conducted respectively (Figure 1C,F,I). Detailed information is listed in Table [Link], [Link], [Link], [Link], [Link], [Link].

### 3.3 Construction of the ceRNA network

**FIGURE 2:**
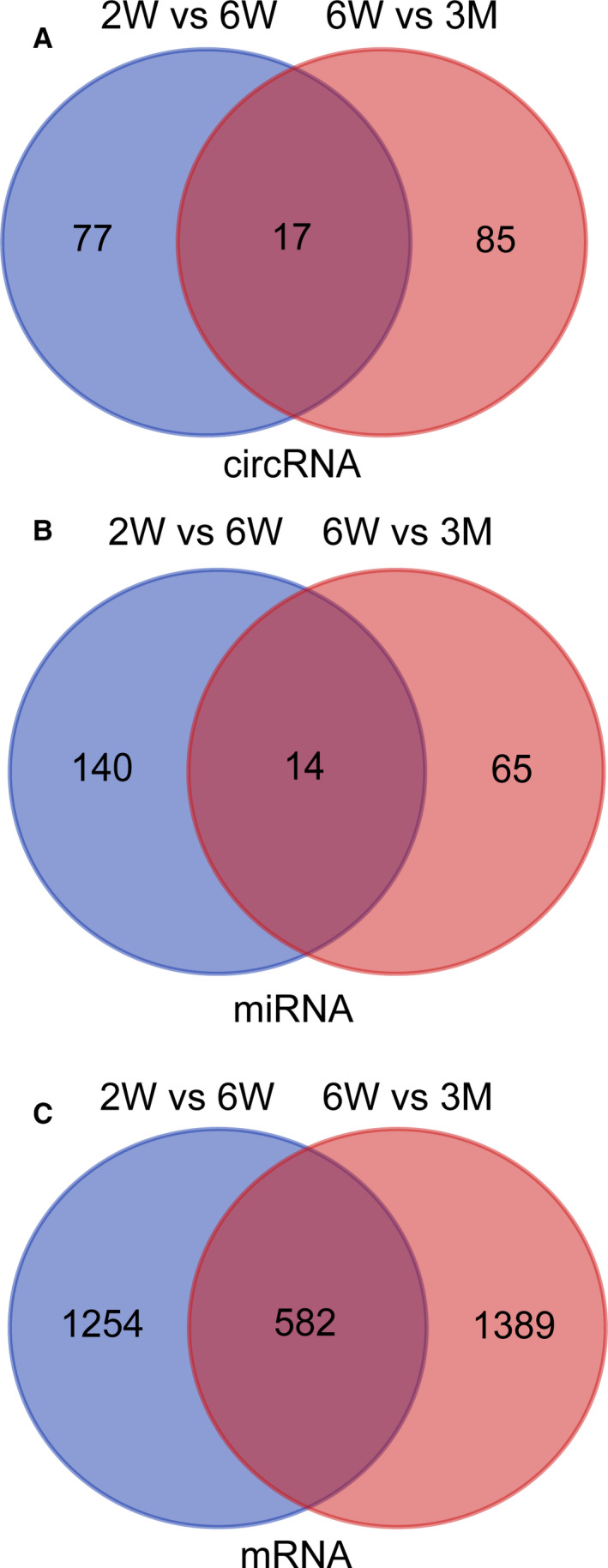
Venn diagrams of differentially expressed transcripts. A, circRNA; B, miRNA; C, mRNA

According to the ceRNA hypothesis, competing endogenous RNAs (ceRNAs) members can regulate each other through competing for the same miRNA response elements (MREs). RNA transcripts communicate with the ceRNA language.^24^ The ceRNA networks in the mouse thymus were constructed based on our RNA‐seq data. As the Venn diagram shown in the Figure 2, we divided the differentially expressed transcripts (circRNAs, miRNAs and mRNAs) into two groups: (a) 2W/6W (+) 6W/3M (−): differential expression in 2W vs 6W group but not in 6W vs 3M group. We consider these transcripts in the group as the factors that play a vital role in the rapid development of the thymus. (b) 2W/6W (−) 6W/3M (+): no differential expression in 2W vs 6W group, but differential expression in 6W vs 3M group. The differential transcripts in this group are thought to be factors that play an important role in the early decline of the thymus.

**FIGURE 3:**
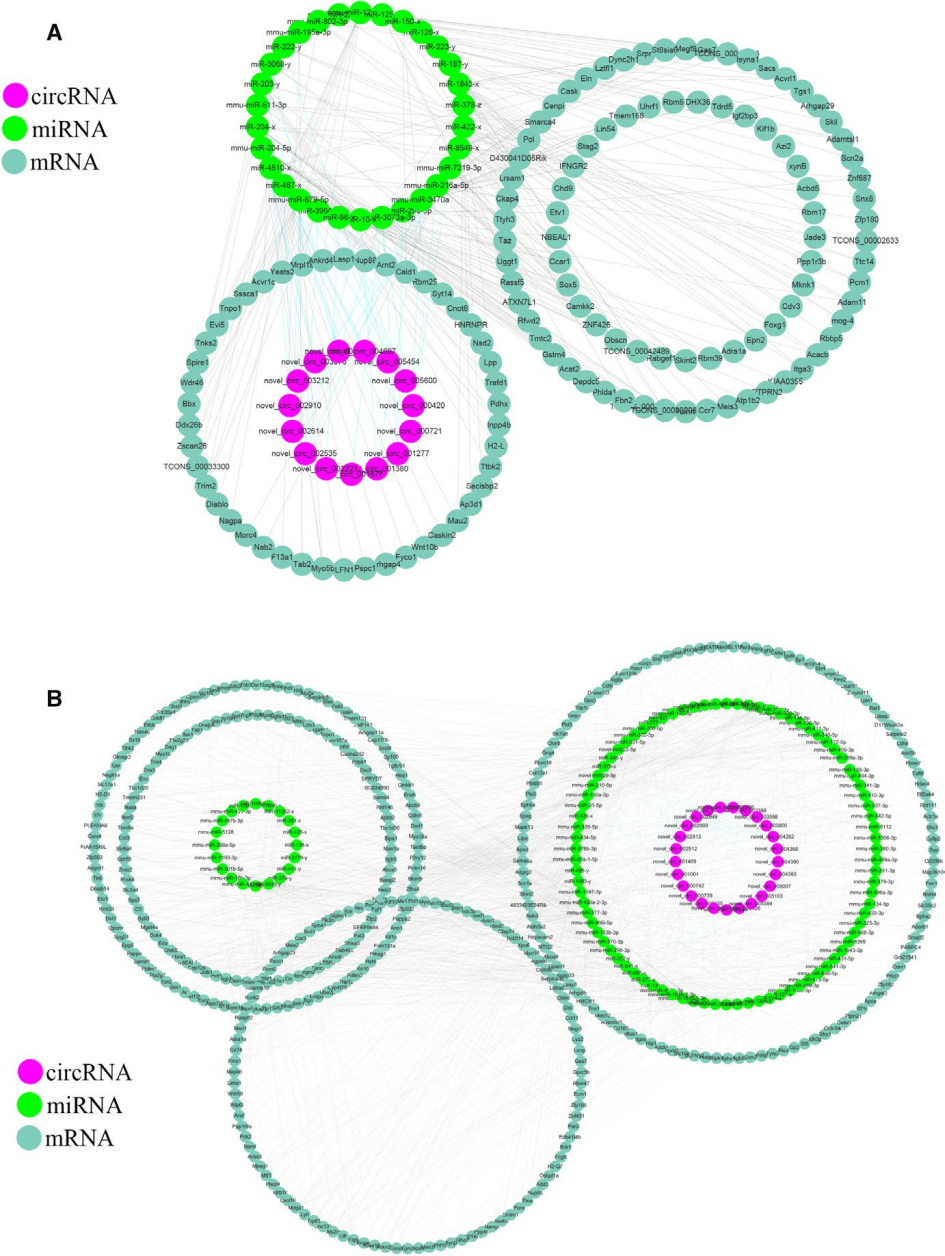
CircRNA‐associated ceRNA networks. CeRNA networks were constructed based on identified circRNA–miRNA and miRNA–mRNA interactions. The networks include increased circRNAs‐decreased miRNAs‐increased mRNAs and decreased circRNAs ‐ increased miRNAs‐decreased mRNAs in 2W/6W (+) and 6W/3M (−) group. (A) down‐up‐down (B) up‐down‐up

**FIGURE 4:**
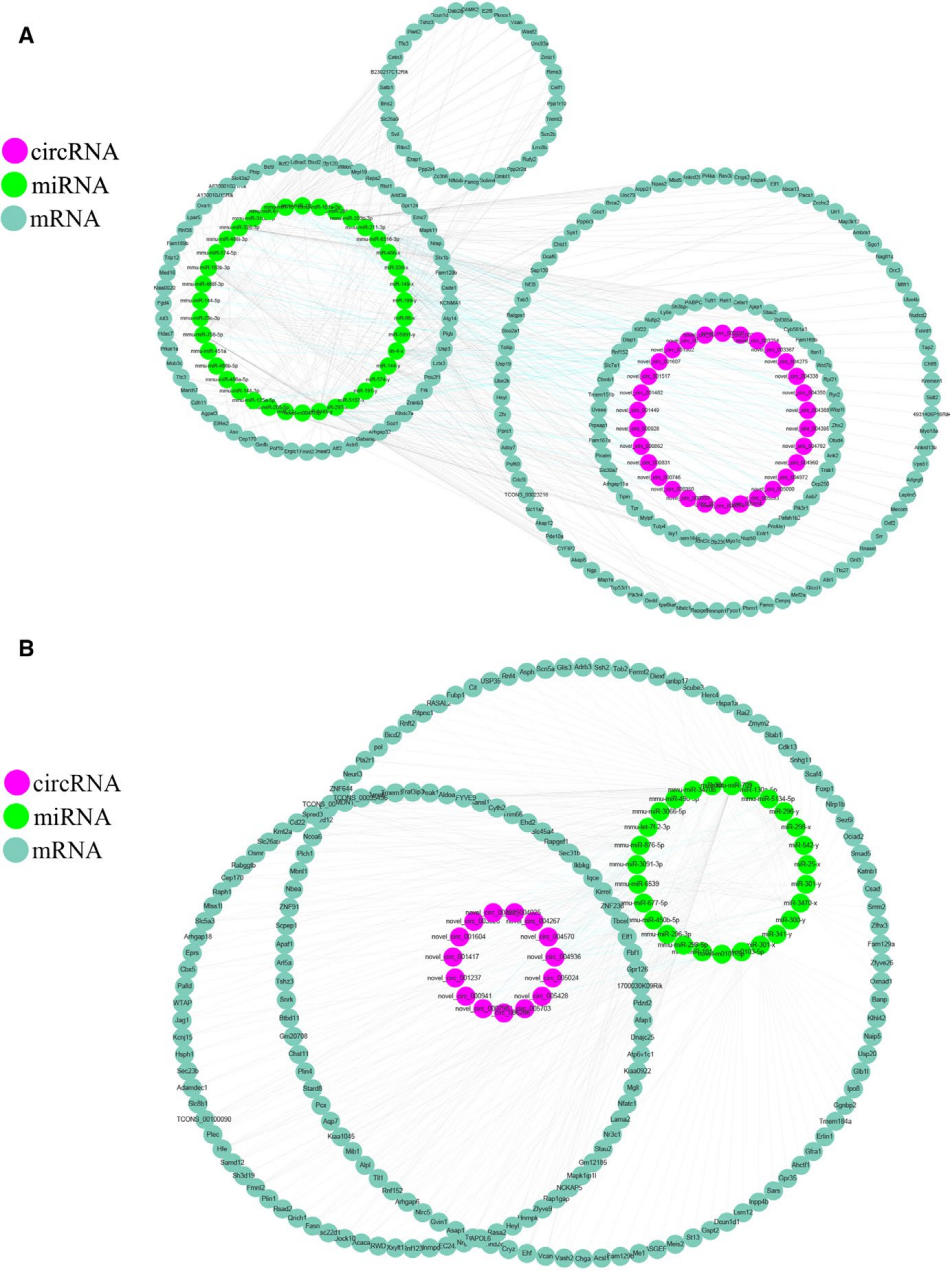
CircRNA‐associated ceRNA networks. CeRNA networks were constructed based on identified circRNA–miRNA and miRNA–mRNA interactions. The networks include increased circRNAs‐decreased miRNAs‐increased mRNAs and decreased circRNAs ‐ increased miRNAs‐decreased mRNAs in 2W/6W (−) and 6W/3M (+) group. (A) down‐up‐down (B) up‐down‐up

A total of 40 circRNAs, 136 miRNAs and 523 mRNAs were significantly dysregulated in the 2W/6W (+) 6W/3M (−) group, which were selected to construct ceRNA networks (Tables S7 and S8). The ceRNA networks included both positive and negative links (Figure 3A,B). Figure 3A shows the decreased circRNAs, increased miRNAs and decreased mRNAs and Figure 3B shows the increased circRNAs, decreased miRNAs and increased mRNAs. In the 2W/6W (−) 6W/3M (+) group, 45 DEcircRNAs, 65 DEmiRNAs and 407 DEmRNAs were used to construct the ceRNA networks (Table S9 and S10). As mentioned above, the ceRNA networks also include two types of links in this group (Figure 4A,B). Figure 4A shows the decreased circRNAs, increased miRNAs and decreased mRNAs and Figure 4B shows the increased circRNAs, decreased miRNAs and increased mRNAs. Information about the differentially expressed transcripts used to construct the networks can be found in Tables [Link], [Link], [Link], [Link]. And the top15 DEcircRNAs between groups was shown in Tables 1 and 2.

**TABLE 1:** Top15 DEcircRNAs in the thymus between 2‐wk‐old and 6‐wk‐old mouse

CircRNA ID	T‐2W_RPM	T‐6W_RPM	log2(FC)	*P* value
novel_circ_002600	6282.377	2524.3654	−1.31539	5.80E‐08
mmu_circ_0000130	1739.733	411.06649	−2.08142	9.51E‐05
novel_circ_002601	3203.865	1161.2558	−1.46413	.000193
novel_circ_002657	637.3261	22.816464	−4.80388	.000193
novel_circ_005640	959.8191	167.44411	−2.51908	.000606
novel_circ_006249	0.001	414.93092	18.66251	.000805
mmu_circ_0000909	0.001	399.84332	18.60908	.001563
novel_circ_002221	596.1799	98.994722	−2.59032	.002377
novel_circ_004311	455.5065	0.001	−18.7971	.002433
novel_circ_002628	0.001	365.43356	18.47925	.002507
novel_circ_002392	0.001	342.61709	18.38624	.003053
novel_circ_004939	3891.912	1720.4442	−1.1777	.003094
novel_circ_001377	412.4453	26.680896	−3.95032	.003707
mmu_circ_0001023	0.001	357.70469	18.44841	.004018
novel_circ_001409	46.89112	517.79007	3.464981	.004374

Abbreviation: FC, fold change; RPM, reads per million mapped reads.

**TABLE 2:** Top15 DEcircRNAs in the thymus between 6‐wk‐old and 3‐mo‐old mouse

CircRNA ID	T‐6W_RPM	T‐3M_RPM	log2(FC)	*P* value
novel_circ_002601	1161.256	4700.284	2.017062	3.25E‐14
novel_circ_002602	1047.173	4015.974	1.939249	2.34E‐09
mmu_circ_0001100	22.81646	944.118	5.37082	4.86E‐07
novel_circ_002600	2524.365	5384.594	1.092917	5.23E‐07
novel_circ_004338	936.5854	97.4279	−3.265	.000121
novel_circ_003454	841.085	64.95194	−3.69481	.000436
novel_circ_001994	247.4868	1066.482	2.107436	.000486
novel_circ_002939	1138.069	219.7915	−2.37238	.000543
novel_circ_004395	521.6545	32.47597	−4.00565	.000775
novel_circ_001902	388.25	0.001	−18.5666	.000947
novel_circ_003367	978.3539	219.7915	−2.15422	.001128
novel_circ_004936	0.001	479.5993	18.87147	.001142
novel_circ_001285	2189.107	981.5215	−1.15725	.001571
mmu_circ_0000861	2337.969	879.166	−1.41105	.001759
novel_circ_004267	22.81646	387.0989	4.084555	.002271

Abbreviations: FC, fold change; RPM, reads per million mapped reads.

### 3.4 Functional enrichment analysis

**FIGURE 5:**
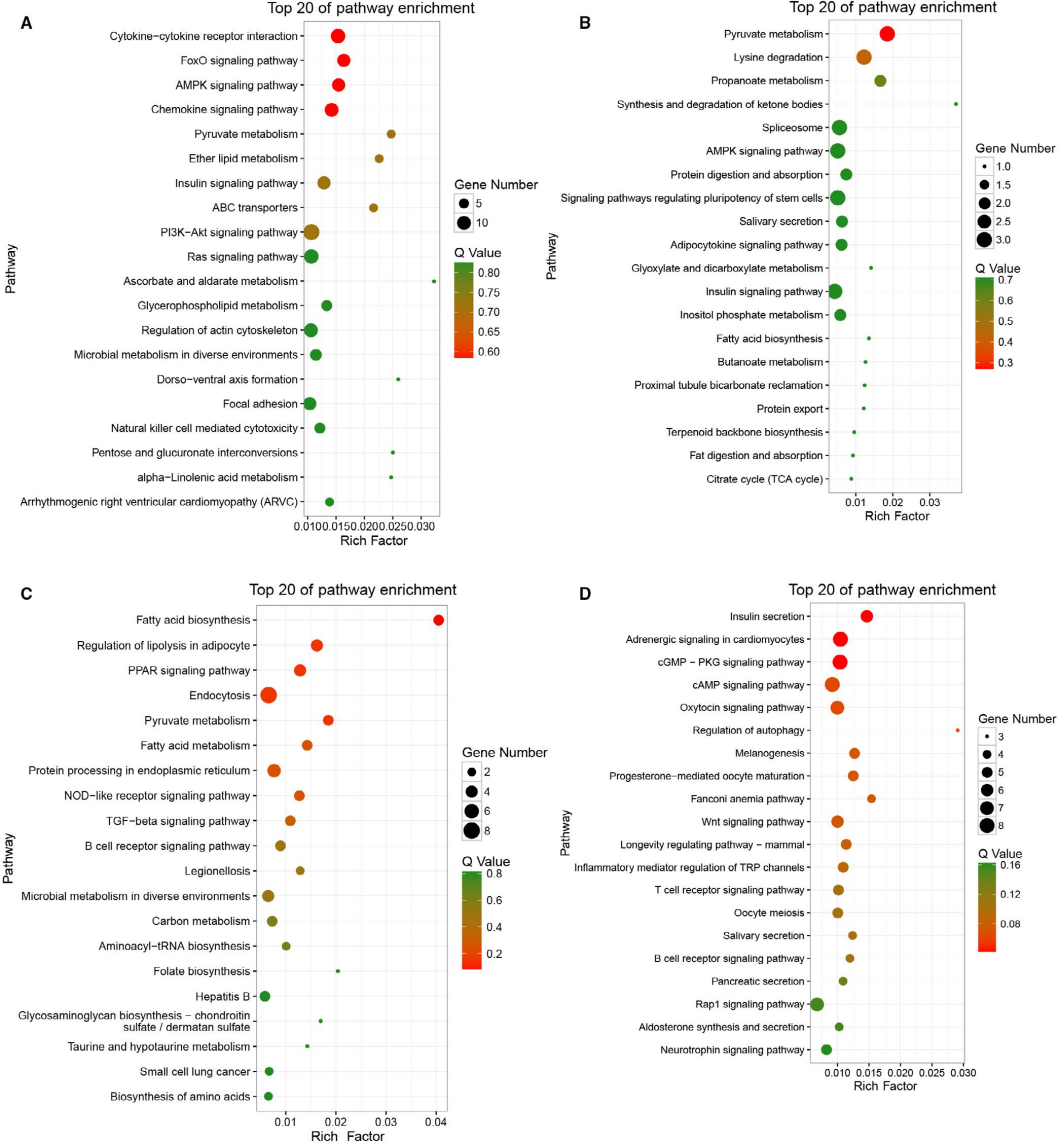
KEGG pathways analysis in the thymus. KEGG pathway enrichment analysis of up and down‐regulated mRNAs with top twenty Enrichment score. (A) 2W/6W (+) 6W/3M (−) group: up‐regulated mRNAs. (B) 2W/6W (+) 6W/3M (−) group: down‐regulated mRNAs. (C) 2W/6W (−) 6W/3M (+) group: up‐regulated mRNAs. (D) 2W/6W (−) 6W/3M (+) group: down‐regulated mRNAs. The *Y*‐axis label represents the pathway and the *X*‐axis label represents the rich factor. The colour and size of the bubble represent enrichment significance and the amount of differentially expressed genes enriched in the pathway, respectively

Kyoto Encyclopedia of Genes and Genomes pathway enrichment analysis was used to determine the underlying biological mechanisms and pathways in the mouse thymus. Bubble charts were used to represent the top 20 pathways in 2W/6W (+) 6W/3M (−) group (Figure 5A,B) and 2W/6W (−) 6W/3M (+) group (Figure 5C,D), respectively. Each group included both positive and negative regulation. In the 2W/6W (+) 6W/3M (−) group, ceRNA gene‐related KEGG pathways were involved in pyruvate metabolism, lysine degradation, PI3K‐Akt signalling pathway, Ras signalling pathway, cytokine‐cytokine receptor interaction, insulin signalling pathway and so on (Tables S11 and 12). The ceRNA genes in the 2W/6W (−) 6W/3M (+) group were associated with biological processes including fatty acid biosynthesis and metabolism, regulation of lipolysis in adipocyte, pyruvate metabolism, insulin secretion, T cell receptor signalling pathway, regulation of autophagy, *etc* (Table S13 and S14).

## 4 DISCUSSION

The thymus is a vital immune organ in humans and other mammals which is the primary site of T cell development.^25^ The thymus provides a specialized environment to facilitate T cell maturation and generate an extremely diverse T cell repertoire. It also establishes its self‐tolerance to prevent autoimmune diseases.^26^ The thymus begins the involution process and apparently exhibits symptoms of age‐related changes at an accelerated rate relative to other organs. Previous studies have found that some miRNAs play an important role in thymic development and involution.^27^, ^28^, ^29^ However, the role of circRNA‐associated ceRNA networks in these processes is unknown. We designed this experiment to further investigate the molecular mechanisms underlining these complex processes of the post‐natal rapid development and subsequent gradual decline in the thymus.

Consistent with the literature,^30^ the mouse thymus undergoes rapid growth after birth. After reaching its maximum size at 6 weeks, the mouse thymus begins to decline. To identify the key molecules involved in these processes and understand their possible mechanisms, RNA‐seq was performed to compare the expression of circRNA, miRNA and mRNA in the thymus of 2‐week‐old, 6‐week‐old and 3‐month‐old mice.

By systematically analysing the sequencing data, we identified DEcircRNAs, DEmiRNAs and DEmRNAs in the 2W vs 6W group and the 6W vs 3M group (fold change ≥2.0 and *P* < .05), and mainly constructed the ceRNA networks of the 2W/6W (+) 6W/3M (–) group and the 2W/6W (−) 6W/3M (+) group. These circRNA‐associated ceRNA networks are potentially involved in the development and early decline of thymus. For instance, mmu_circ_0001023, novel_circ_003800 were identified as ceRNAs of mmu‐miR‐434‐5p, which targets *Ids*. The expression of *Ids* was higher in 6‐week‐old mice than that in 2‐week‐old mice. *Ids* are necessary for skeletal myogenesis,^31^ cardiac development^32^ and also play key roles in the differentiation and proliferation of T cells.^33^, ^34^ Moreover, mmu_circ_0000909 was found to be a ceRNA of mmu‐miR‐92a‐1‐5p, which targets *KMT2D* *KMT2D* is a major enhancer of H3K4me1/2 methyltransferase which is essential for early embryonic development in mice.^35^ There is evidence that *KMT2D* is required for cell‐type‐specific gene expression and cell differentiation in model systems for adipogenesis and myogenesis.^36^ Its mutation is closely related to the occurrence of T and B cell‐associated lymphoma.^37^, ^38^ In the 2W/6W (−) 6W/3M (+) group, mmu_circ_0000459, novel_circ_000862, novel_circ_001902 and novel_circ_002116 were identified as ceRNAs of mmu‐miR‐125a‐5p, which targets *Erap1*. *Erap1* is an M1 zinc metalloprotease family member which has a significant impact on peptide processing function and the repertoire of peptides presented. In addition, the deregulation of *ERAP1* can affect CD8^+^ T cell response.^39^, ^40^ Several researches have shown that the absence of *ERAP1* expression in mice results in increased production of proinflammatory cytokines.^39^ The occurrence of inflammation is closely related to thymic ageing.^4^, ^41^ The down‐regulation of *Erap1* in 3‐month‐old mice may be associated with the early thymic decline. Besides, mmu_circ_0001431, novel_circ_004960, novel_circ_005000 and novel_circ_005893 were identified as ceRNAs of mmu‐miR‐135a‐5p, which targets *Celf1*. *Celf1*, a member of the *Celf* family, belongs to RNA‐binding proteins. Previous research has indicated that *Celf1* may affect the embryonic development process^42^, ^43^ and involve in the proliferation and division of T cells.^44^ Other links in these ceRNA networks are listed in Tables [Link], [Link], [Link], [Link].

In order to clarify ceRNA functions in mouse thymus, KEGG pathway enrichment analysis was used to determine the underlying biological pathways. The metabolism pathways such as pyruvate metabolism, fatty acid metabolism were significantly enriched pathways in both groups. Metabolism interconnects with signalling events to influence cell cycle, differentiation, cell death and immunological function.^45^ Yang *et al* ^46^ reported that mTORC1 coordinated multiple metabolic programmes in T cells including glycolysis, lipid synthesis and oxidative phosphorylation to affect lymphocyte activation and fate decisions in adaptive immunity. In addition, Ras signalling pathway was found to be enriched in the 2W/6W (+) 6W/3M (−) group. Some evidence indicates that the activation of the Ras signalling pathway is critical for T cell development in the thymus.^47^, ^48^, ^49^ In the 2W/6W (−) 6W/3M (+) group, the expression of DEmRNAs related to the Insulin secretion pathway was significantly down‐regulated. Insulin is a key hormone which controls the metabolism of carbohydrates, proteins and lipids. Previous studies indicated that insulin receptor signalling controlled T cell proliferation, cytokine production and regulated adaptive immunity.^50^, ^51^ It suggests that insulin signalling pathway may modulate the thymus ageing through controlling the development of the thymocytes.

In summary, we demonstrated the circRNA‐associated ceRNA profiles of the mouse thymus at different time points by using deep‐RNA‐seq analysis for the first time. These results shed light on the possible contribution of the ceRNAs to the development and involution of the thymus.

## CONFLICT OF INTEREST

The authors declare that there are no conflicts of interest.

## AUTHORS CONTRIBUTIONS

WZ, JW and YZ designed the study. WL, NM, BY, YZ, YY, QL and T.YW. participated in the animal experiments including tissue collection and RNA/protein extraction. WL, NM, XF.C., JW and WZ analysed the data and wrote the paper. The final manuscript has been seen and approved by all authors.

## Supporting information

Fig S1

Table S1

Table S2

Table S3

Table S4

Table S5

Table S6

Table S7

Table S8

Table S9

Table S10

Table S11

Table S12

Table S13

Table S14

Table S15

## References

[1] Parkin J , Cohen B . An overview of the immune system. Lancet (London, England). 2001;357:1777‐1789. 10.1016/S0140-6736(00)04904-7 11403834

[2] Wu H , Qin X , Dai H , Zhang Y . Time‐course transcriptome analysis of medullary thymic epithelial cells in the early phase of thymic involution. Mol Immunol. 2018;99:87‐94. 29730547 10.1016/j.molimm.2018.04.010

[3] Zaharie D , Moleriu RD , Mic FA . Modeling the development of the post‐natal mouse thymus in the absence of bone marrow progenitors. Sci Rep. 2016;6:36159. 27824070 10.1038/srep36159 PMC5099910

[4] Ye Y , Li D , Ouyang D , et al. MicroRNA expression in the aging mouse thymus. Gene. 2014;547:218‐225. 24956559 10.1016/j.gene.2014.06.039

[5] Guo Z , Chi F , Song Y , et al. Transcriptome analysis of murine thymic epithelial cells reveals ageassociated changes in microRNA expression. Int J Mol Med. 2013;32:835‐842. 23969555 10.3892/ijmm.2013.1471

[6] Zhang H , Artiles KL , Fire AZ . Functional relevance of "seed" and "non‐seed" sequences in microRNA‐mediated promotion of *C elegans* developmental progression. RNA (New York, NY). 2015;21:1980‐1992. 10.1261/rna.053793.115 PMC4604436 26385508

[7] Constantin L , Constantin M , Wainwright BJ . MicroRNA biogenesis and hedgehog‐patched signaling cooperate to regulate an important developmental transition in granule cell development. Genetics. 2016;202:1105‐1118. 26773048 10.1534/genetics.115.184176 PMC4788112

[8] Li Y , Chen D , Jin L , et al. MicroRNA‐20b‐5p functions as a tumor suppressor in renal cell carcinoma by regulating cellular proliferation, migration and apoptosis. Mol Med Rep. 2016;13:1895‐1901. 26708577 10.3892/mmr.2015.4692

[9] Li Y , Chen D , Jin LU , et al. Oncogenic microRNA‐142‐3p is associated with cellular migration, proliferation and apoptosis in renal cell carcinoma. Oncol Lett. 2016;11:1235‐1241. 26893725 10.3892/ol.2015.4021 PMC4734216

[10] Geiger J , Dalgaard LT . Interplay of mitochondrial metabolism and microRNAs. Cell Mol Life Sci. 2017;74:631‐646. 27563705 10.1007/s00018-016-2342-7 PMC11107739

[11] Xu M , Gan T , Ning H , Wang L . MicroRNA functions in thymic biology: thymic development and involution. Front Immunol. 2018;9:2063. 30254640 10.3389/fimmu.2018.02063 PMC6141719

[12] Kroesen BJ , Teteloshvili N , Smigielska‐Czepiel K , et al. Immuno‐miRs: critical regulators of T‐cell development, function and ageing. Immunology. 2015;144:1‐10. 25093579 10.1111/imm.12367 PMC4264905

[13] Berrih‐Aknin S , Le Panse R . Myasthenia gravis: a comprehensive review of immune dysregulation and etiological mechanisms. J Autoimmun. 2014;52:90‐100. 24389034 10.1016/j.jaut.2013.12.011

[14] Guo D , Ye Y , Qi J , et al. MicroRNA‐195a‐5p inhibits mouse medullary thymic epithelial cells proliferation by directly targeting Smad7. Acta Biochim Biophys Sin. 2016;48:290‐297. 26837421 10.1093/abbs/gmv136 PMC4885129

[15] Qu S , Liu Z , Yang X , et al. The emerging functions and roles of circular RNAs in cancer. Cancer Lett. 2018;414:301‐309. 29174799 10.1016/j.canlet.2017.11.022

[16] Kristensen LS , Andersen MS , Stagsted LVW , et al. The biogenesis, biology and characterization of circular RNAs. Nature reviews Genetics. 2019; 20: 675‐691. 10.1038/s41576-019-0158-7 31395983

[17] Yang W , Du WW , Li X , Yee AJ , Yang BB . Foxo3 activity promoted by non‐coding effects of circular RNA and Foxo3 pseudogene in the inhibition of tumor growth and angiogenesis. Oncogene. 2016;35:3919‐3931. 26657152 10.1038/onc.2015.460

[18] Han D , Li J , Wang H , et al. Circular RNA circMTO1 acts as the sponge of microRNA‐9 to suppress hepatocellular carcinoma progression. Hepatology (Baltimore, MD). 2017;66:1151‐1164. 10.1002/hep.29270 28520103

[19] Lukiw WJ , Circular RNA . circRNA) in Alzheimer's disease (AD. Front Genet. 2013;4:307. 24427167 10.3389/fgene.2013.00307 PMC3875874

[20] Wang YS , Zhou J , Hong K , Cheng XS , Li YG . MicroRNA‐223 displays a protective role against cardiomyocyte hypertrophy by targeting cardiac troponin I‐interacting kinase. Cell Physiol Biochem. 2015;35:1546‐1556. 25792377 10.1159/000373970

[21] Zhang C , Wang X , Chen Y , Wu Z , Zhang C , Shi W . The down‐regulation of hsa_circ_0012919, the sponge for miR‐125a‐3p, contributes to DNA methylation of CD11a and CD70 in CD4(+) T cells of systemic lupus erythematous. Clin Sci (London, England. 1979;2018(132):2285‐2298. 10.1042/CS20180403 30237316

[22] Fuchs S , Naderi J , Meggetto F . Non‐coding RNA networks in ALK‐positive anaplastic‐large cell lymphoma. Int J Mol Sci. 2019;20(9):2150. 10.3390/ijms20092150 PMC6539248 31052302

[23] Rong D , Sun H , Li Z , et al. An emerging function of circRNA‐miRNAs‐mRNA axis in human diseases. Oncotarget. 2017;8:73271‐73281. 29069868 10.18632/oncotarget.19154 PMC5641211

[24] Shu X , Cheng L , Dong Z , Shu S . Identification of circular RNA‐associated competing endogenous RNA network in the development of cleft palate. J Cell Biochem. 2019;120:16062‐16074. 31074068 10.1002/jcb.28888

[25] Petrie HT , Zuniga‐Pflucker JC . Zoned out: functional mapping of stromal signaling microenvironments in the thymus. Annu Rev Immunol. 2007;25:649‐679. 17291187 10.1146/annurev.immunol.23.021704.115715

[26] Pearse G . Normal structure, function and histology of the thymus. Toxicol Pathol. 2006;34:504‐514. 17067941 10.1080/01926230600865549

[27] Ebert PJ , Jiang S , Xie J , Li QJ , Davis MM . An endogenous positively selecting peptide enhances mature T cell responses and becomes an autoantigen in the absence of microRNA miR‐181a. Nat Immunol. 2009;10:1162‐1169. 19801983 10.1038/ni.1797 PMC3762483

[28] Zheng Q , Zhou L , Mi QS . MicroRNA miR‐150 is involved in Valpha14 invariant NKT cell development and function. J Immunol (Baltimore, Md. 1950;2012(188):2118‐2126. 10.4049/jimmunol.1103342 PMC7375412 22287707

[29] Xu M , Sizova O , Wang L , Su DM . A fine‐tune role of Mir‐125a‐5p on Foxn1 during age‐associated changes in the thymus. Aging Dis. 2017;8:277‐286. 28580184 10.14336/AD.2016.1109 PMC5440108

[30] Shanley DP , Aw D , Manley NR , Palmer DB . An evolutionary perspective on the mechanisms of immunosenescence. Trends Immunol. 2009;30:374‐381. 19541538 10.1016/j.it.2009.05.001

[31] Biederer CH , Ries SJ , Moser M , et al. The basic helix‐loop‐helix transcription factors myogenin and Id2 mediate specific induction of caveolin‐3 gene expression during embryonic development. J Biol Chem. 2000;275:26245‐26251. 10835421 10.1074/jbc.M001430200

[32] Springhorn JP , Ellingsen O , Berger HJ , Kelly RA , Smith TW . Transcriptional regulation in cardiac muscle. Coordinate expression of Id with a neonatal phenotype during development and following a hypertrophic stimulus in adult rat ventricular myocytes in vitro. J Biol Chem. 1992;267:14360‐14365. 1378442

[33] Maruyama T , Li J , Vaque JP , et al. Control of the differentiation of regulatory T cells and T(H)17 cells by the DNA‐binding inhibitor Id3. Nat Immunol. 2011;12:86‐95. 21131965 10.1038/ni.1965 PMC3140164

[34] Nakatsukasa H , Zhang D , Maruyama T , et al. The DNA‐binding inhibitor Id3 regulates IL‐9 production in CD4(+) T cells. Nat Immunol. 2015;16:1077‐1084. 26322481 10.1038/ni.3252 PMC5935106

[35] Wang C , Lee JE , Lai B , et al. Enhancer priming by H3K4 methyltransferase MLL4 controls cell fate transition. Proc Natl Acad Sci USA. 2016;113:11871‐11876. 27698142 10.1073/pnas.1606857113 PMC5081576

[36] Lee JE , Wang C , Xu S , et al. H3K4 mono‐ and di‐methyltransferase MLL4 is required for enhancer activation during cell differentiation. eLife. 2013;2:e01503. 24368734 10.7554/eLife.01503 PMC3869375

[37] Zhang J , Dominguez‐Sola D , Hussein S , et al. Disruption of KMT2D perturbs germinal center B cell development and promotes lymphomagenesis. Nat Med. 2015;21:1190‐1198. 26366712 10.1038/nm.3940 PMC5145002

[38] Ji MM , Huang YH , Huang JY , et al. Histone modifier gene mutations in peripheral T‐cell lymphoma not otherwise specified. Haematologica. 2018;103:679‐687. 29305415 10.3324/haematol.2017.182444 PMC5865443

[39] Aldhamen YA , Seregin SS , Rastall DP , et al. Endoplasmic reticulum aminopeptidase‐1 functions regulate key aspects of the innate immune response. PLoS ONE. 2013;8:e69539. 23894499 10.1371/journal.pone.0069539 PMC3722114

[40] Lopez de Castro JA . How ERAP1 and ERAP2 shape the peptidomes of disease‐associated MHC‐I proteins. Front Immunol. 2018;9:2463. 30425713 10.3389/fimmu.2018.02463 PMC6219399

[41] Yue S , Zheng X , Zheng Y . Cell‐type‐specific role of lamin‐B1 in thymus development and its inflammation‐driven reduction in thymus aging. Aging Cell. 2019;18:e12952. 30968547 10.1111/acel.12952 PMC6612680

[42] Zheng Y , Miskimins WK . CUG‐binding protein represses translation of p27Kip1 mRNA through its internal ribosomal entry site. RNA Biol. 2011;8:365‐371. 21508681 10.4161/rna.8.3.14804 PMC3218506

[43] Zhang L , Lee JE , Wilusz J , Wilusz CJ . The RNA‐binding protein CUGBP1 regulates stability of tumor necrosis factor mRNA in muscle cells: implications for myotonic dystrophy. J Biol Chem. 2008;283:22457‐22463. 18559347 10.1074/jbc.M802803200 PMC2504872

[44] Beisang D , Reilly C , Bohjanen PR . Alternative polyadenylation regulates CELF1/CUGBP1 target transcripts following T cell activation. Gene. 2014;550:93‐100. 25123787 10.1016/j.gene.2014.08.021 PMC4162518

[45] Wang R , Green DR . Metabolic checkpoints in activated T cells. Nat Immunol. 2012;13:907‐915. 22990888 10.1038/ni.2386

[46] Yang K , Shrestha S , Zeng H , et al. T cell exit from quiescence and differentiation into Th2 cells depend on Raptor‐mTORC1‐mediated metabolic reprogramming. Immunity. 2013;39:1043‐1056. 24315998 10.1016/j.immuni.2013.09.015 PMC3986063

[47] Lapinski PE , Qiao Y , Chang CH , King PD . A role for p120 RasGAP in thymocyte positive selection and survival of naive T cells. J Immunol (Baltimore, Md. 1950;2011(187):151‐163. 10.4049/jimmunol.1100178 PMC3119767 21646295

[48] Kortum RL , Rouquette‐Jazdanian AK , Samelson LE . Ras and extracellular signal‐regulated kinase signaling in thymocytes and T cells. Trends Immunol. 2013;34:259‐268. 23506953 10.1016/j.it.2013.02.004 PMC3856398

[49] Iborra S , Soto M , Stark‐Aroeira L , et al. H‐ras and N‐ras are dispensable for T‐cell development and activation but critical for protective Th1 immunity. Blood. 2011;117:5102‐5111. 21444916 10.1182/blood-2010-10-315770

[50] Fischer HJ , Sie C , Schumann E , et al. The insulin receptor plays a critical role in T cell function and adaptive immunity. J Immunol (Baltimore, Md. 1950;2017(198):1910‐1920. 10.4049/jimmunol.1601011 28115529

[51] Tsai S , Clemente‐Casares X , Zhou AC , et al. Insulin receptor‐mediated stimulation boosts T cell immunity during inflammation and infection. Cell Metab. 2018;28:922‐934.e4. 30174303 10.1016/j.cmet.2018.08.003

