# Supplementary figures and images for: Comprehensive analysis of circRNA expression profiles and circRNA‐associated competing endogenous RNA networks in the development of mouse thymus
Source: J Cell Mol Med. 2020 Apr 19;24(11):6340–9. doi: 10.1111/jcmm.15276 (PMC7294154; doi:10.1111/jcmm.15276)

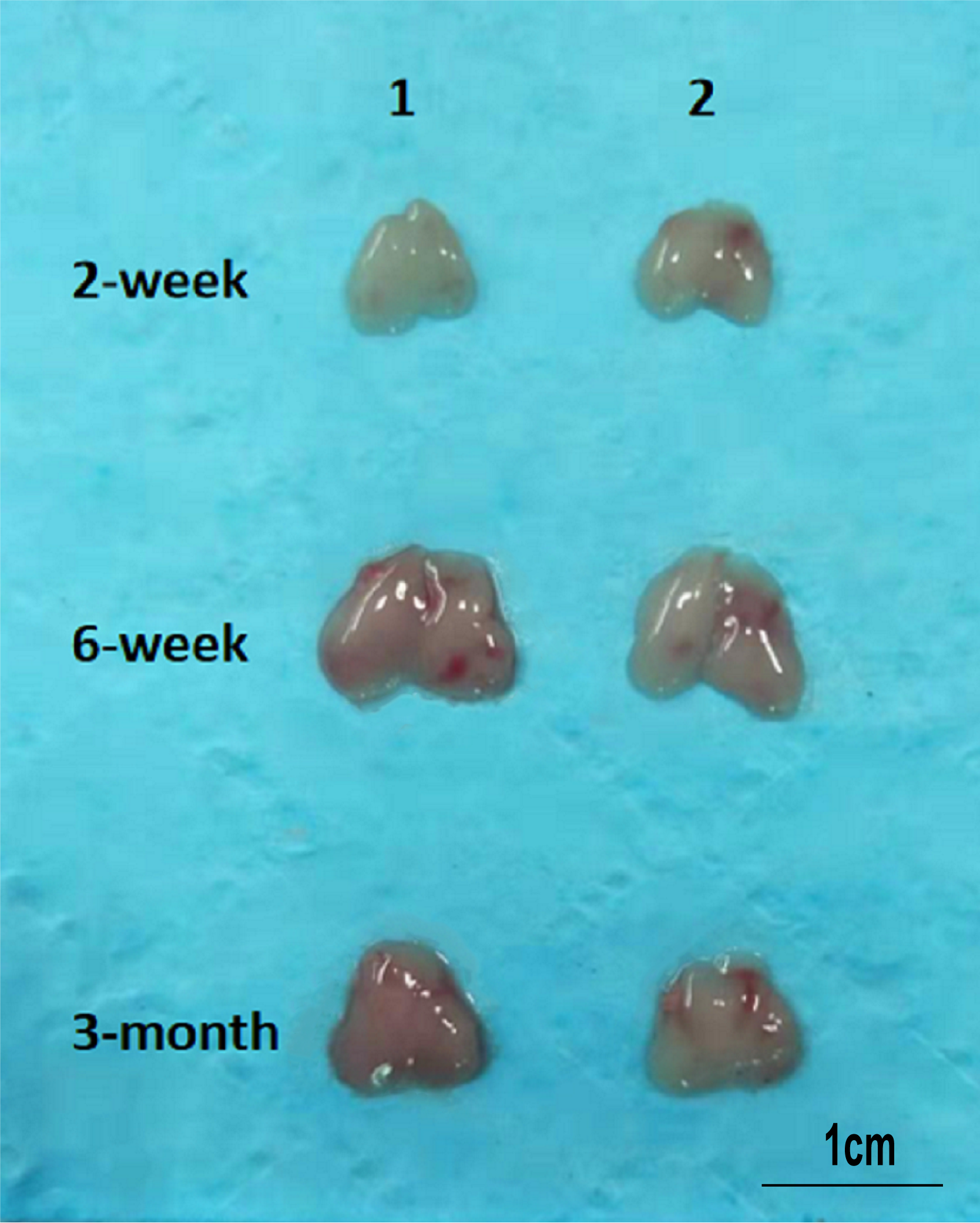

Supplement: Supplementary file 1 — Fig S1 [file JCMM-24-6340-s001.tif]
