# Supplementary material for: Comprehensive analysis of circRNA expression profiles and circRNA‐associated competing endogenous RNA networks in the development of mouse thymus
Source: J Cell Mol Med. 2020 Apr 19;24(11):6340–9. doi: 10.1111/jcmm.15276 (PMC7294154; doi:10.1111/jcmm.15276)
Supplement: Supplementary file 16 — Table S15 [file JCMM-24-6340-s016.docx]

**RNA-seq**

After total RNA was extracted, rRNAs were removed to retain mRNAs and ncRNAs. The enriched mRNAs and ncRNAs were fragmented into short fragments by using fragmentation buffer and reverse transcribed into cDNA with random primers. Second-strand cDNA were synthesized by DNA polymerase I, RNase H, dNTP (dUTP instead of dTTP) and buffer. Next, the cDNA fragments were purified with QiaQuick PCR extraction kit, end repaired, poly(A) added, and ligated to Illumina sequencing adapters. Then UNG (Uracil-N-Glycosylase) was used to digest the second-strand cDNA. The digested products were size selected by agarose gel electrophoresis, PCR amplified, and sequenced using Illumina HiSeqTM 4000 by Gene Denovo Biotechnology Co. (Guangzhou, China).

**miRNA-seq**

After total RNA was extracted by Trizol, the RNA molecules in a size range of 18–30nt were enriched by polyacrylamide gel electrophoresis (PAGE). Then the 3’ adapters were added and the 36-44nt RNAs were enriched. The 5’ adapters were then ligated to the RNAs as well. The ligation products were reverse transcribed by PCR amplification and the 140-160bp size PCR products were enriched to generate a cDNA library and sequenced using Illumina HiSeqTM 2500 by Gene Denovo Biotechnology Co. (Guangzhou, China).
